# You are what you eat: metabolic recycling of phagocytosed bacteria modulates macrophage immunity

**DOI:** 10.1038/s41392-025-02222-x

**Published:** 2025-04-28

**Authors:** Syamantak Basu, Manuela Rossol

**Affiliations:** 1 Molecular Immunology, Faculty of Health Sciences, BTU Cottbus-Senftenberg, Senftenberg, Germany; 2 Faculty of Environment and Natural Sciences, BTU Cottbus-Senftenberg, Senftenberg, Germany

**Keywords:** Infection, Cell biology, Innate immune cells

In a recent study published in *Nature*, Lesbats et al.^1^ show that phagocytosed bacteria can be recycled by macrophages to serve as an alternative nutrient source. Bacterial cAMP was identified as a signal for macrophages to differentiate between dead and live bacteria and to adjust the inflammatory response accordingly.

You are what you eat—at least when it comes to macrophages. Macrophages have a high capacity to take up fluid, macromolecules, apoptotic cells, and microorganisms from the extracellular space. Uptake of macromolecules by macropinocytosis and subsequent degradation in lysosomes in an infectious or autoimmune setting can lead to excessive inflammation.^2^ It is also known that engulfment of apoptotic cells and digestion in phagolysosomes by the macrophages generates amino acids, nucleotides, and fatty acids that then act as metabolites and signalling molecules in the macrophages and influence their cellular functions.^3^ Up until now it was largely believed that phagolysosomes containing engulfed microorganisms are tightly sealed vesicles, so that potentially harmful components from the foreign organisms aren’t diffused in the cell. Lesbats et al. show here for the first time that macrophages recycle engulfed bacteria and use the components as nutrient source.

The authors showed that amino acids supplied by lysosomal degradation of dead bacteria (*Escherichia coli*) powers mitochondrial metabolism. The fate of the carbon atoms of ^13^C-labelled dead bacteria in macrophages was traced and it was found that various metabolites were labelled, most importantly itaconate, which is a macrophage specific metabolite. The results were also verified in vivo and with other bacterial species and the authors confirmed that phagocytosis of dead bacteria act as a nutrient source by providing intermediates which can be recycled.

The study also delved into the signalling mechanisms behind this process. The mechanistic target of rapamycin complex C1 (mTORC1) is a central nutrient sensing complex which senses amino acids in the lysosomes and modulates metabolic efflux. mTORC1 is recruited to the lysosomes by Rag GTPases and Rheb GTPases stimulate its kinase activity. The authors used macrophages from transgenic *RagA*^*GTP/Δ*^ mice that have a constitutively activated mTORC1 pathway via a GTP-bound version of RagA. Recycling of bacterial-derived amino acids was suppressed by mTORC1 activation, reducing their incorporation into macrophage metabolism, as observed by a decreased ^13^C incorporation. In contrast, macrophages from RagA-deficient mice showed an increased ^13^C incorporation.

The viability of the engulfed bacteria was also shown to be relevant in recycling of bacterial metabolites, which was more efficient when dead bacteria were taken up. Live and highly replicating bacteria would compete with host cells for nutrients during a microbial infection and this would create a nutrient-restricting microenvironment.^4^ The authors simulated this microenvironment by amino acid deprivation. Phagocytosis of live bacteria under these conditions led to cell death and an enhanced production of reactive oxygen species (ROS). Live bacteria also induce pro-inflammatory IL-1β, and ROS are potent inducers. Phagocytosis of dead bacteria led to bacteria-derived ^13^C incorporation in itaconate and ^13^C enrichment in amino acids involved in the glutathione biosynthetic pathway, indicating that the process decreased ROS production by supplying metabolic intermediates necessary for anti-oxidant responses. Consequently, amino acid supplementation in macrophages that engulfed live bacteria limited the IL-1β production. The authors conclude that live and dead bacteria are a source of nutrients for macrophages following phagocytosis, but dead bacteria are recycled more efficiently.

It makes sense for the macrophage to differentiate between live and dead bacteria. If the bacteria in the extracellular space are dead, i.e., the immune response was already successful, continuing the pro-inflammatory insult would be harmful to the host. But how does the macrophage recognise live or dead bacteria? It was previously reported that cyclic-di-adenosine monophosphate (c-di-AMP), a bacterial second messenger present in live Gram-positive bacteria, is such a signal which can be detected by macrophages.^5^ Here, the authors performed a metabolomic analysis of live and dead bacteria, and found a striking accumulation of 3’,5’-cyclic adenosine monophosphate (cAMP) in dead bacteria, and ^13^C labelled AMP. AMP is the main product of cAMP hydrolysis and upon phagocytosis of dead bacteria, bacterial cAMP is hydrolysed in the macrophages to form AMP and its intermediates. The alteration of the ATP:AMP ratio then triggers AMP-dependent signalling in macrophages. AMP-activated protein kinase (AMPK), which inhibits the mTOR pathway, was activated by phagocytosis of dead bacteria. The authors also noted that phagocytosis of live bacteria decreased the AMP pool in macrophages. AMP, derived from hydrolysis of bacterial cAMP in macrophages after phagocytosis of dead bacteria, can therefore modulate macrophage metabolism by activating AMPK and inhibiting mTORC1.

The discovery by Lesbats et al. is ground-breaking as it proves that macrophages do not only destroy bacteria by phagocytosis but also reuse them to fuel their own metabolic needs including protein synthesis and mitochondrial oxidative phosphorylation. And in the context of ‘you are what you eat’, the authors demonstrated that when macrophages eat live bacteria it triggers an inflammatory response, while when the macrophages eat dead bacteria this potential disease-driving, and at this point unnecessary response is not triggered. They identified mTORC1 as an important switch regulating the inflammation response of macrophages and cAMP as a signal for the macrophages to identify dead bacteria and adjust the metabolism and inflammatory response (Fig. 1). This ability of the macrophages can be beneficial in formulating new anti-infective drugs and vaccines by conditioning the macrophages at bacterial infection sites. Additionally, understanding cellular switches between inflammation and resolution of inflammation and targeting metabolic pathways like mTORC1 may offer strategies to control excessive inflammation observed in various diseases, including chronic inflammatory conditions and sepsis. In summary, the study by Lesbats et al. shows that modulating macrophage metabolism via mTORC1 or bacterial-derived metabolic signals could offer novel strategies for immunotherapy and infectious disease treatment.

**Fig. 1:**
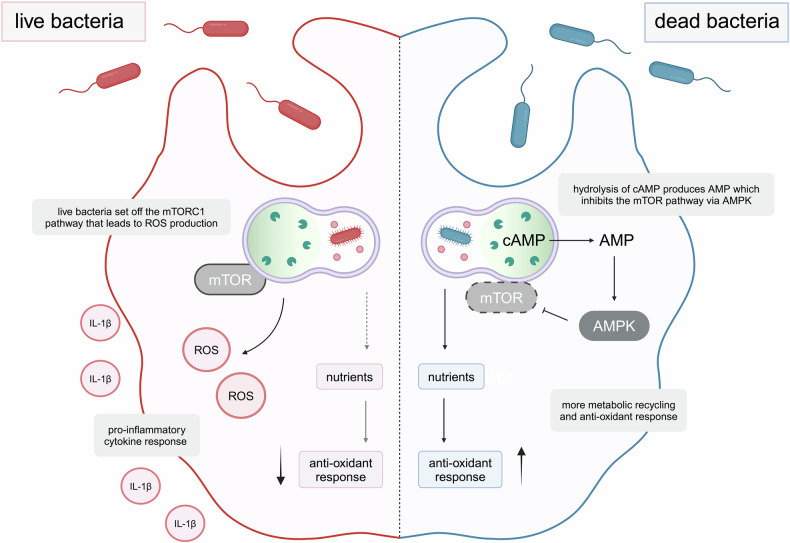
Phagocytosis of live bacteria sets off the mTORC1 pathway leading to high production of reactive oxygen species (ROS) and consequently pro-inflammatory cytokines such as IL-1β (left in red). Dead bacteria, however, can be recognised by cAMP accumulation which on hydrolysis produces AMP. This inhibits the mTORC1 pathway via AMPK, leading to higher recycling of bacterial nutrients for synthesis of metabolites required by the cell, most importantly metabolites involved in the antioxidant response (right in blue). The figure was created using BioRender

## References

[1] Lesbats, J. et al. Macrophages recycle phagocytosed bacteria to fuel immunometabolic responses. *Nature*. 10.1038/s41586-025-08629-4 (2025). 10.1038/s41586-025-08629-4 40011782

[2] Jäger, E. et al. Calcium-sensing receptor-mediated NLRP3 inflammasome response to calciprotein particles drives inflammation in rheumatoid arthritis. *Nat. Commun.* **11**, 4243 (2020). 32843625 10.1038/s41467-020-17749-6 PMC7447633

[3] Schilperoort, M. et al. The role of efferocytosis-fueled macrophage metabolism in the resolution of inflammation. *Immunol. Rev.* **319**, 65–80 (2023). 37158427 10.1111/imr.13214 PMC10615666

[4] Tucey, T. M. et al. Metabolic competition between host and pathogen dictates inflammasome responses to fungal infection. *PLoS Pathog.* **16**, e1008695 (2020). 32750090 10.1371/journal.ppat.1008695 PMC7433900

[5] Moretti, J. et al. STING senses microbial viability to orchestrate stress-mediated autophagy of the endoplasmic reticulum. *Cell* **171**, 809–823.e13 (2017). 29056340 10.1016/j.cell.2017.09.034 PMC5811766

